# Supplementary material for: Scientific discovery in a model-centric framework: Reproducibility, innovation, and epistemic diversity
Source: PLoS One. 2019 May 15;14(5):e0216125. doi: 10.1371/journal.pone.0216125 (PMC6519896; doi:10.1371/journal.pone.0216125)
Supplement: S1 Algorithm — (PDF) [file pone.0216125.s001.pdf]

Agent-based model algorithm.

We first present the general algorithm for stochastic process of scientific discovery with replicator in the agent-based model and then discuss specific values used in the article.

1: Input: $\mathcal{M}, \Theta, S, \mathcal{R}, \mathbb{P}(R_a), \mathbb{P}(M|R_a, M_G^{(t)}), M_G^{(0)}, M_T, \theta_T, n, t_{max}$
2: Set $t = 0$
3: **while** $t < t_{max}$ **do**
4:     Simulate $R_a \sim \text{Categorical}(p_1, p_2, \cdots, p_A)$
5:     Simulate $M_P^{(t)} \sim \mathbb{P}(M|R_a, M_G^{(t)})$
6:     Simulate $D_i^{(t)} \sim M_T(\theta_T)$, for $i = 1, 2, \cdots, n$ independently of each other
7:     Calculate

$$S(M_P^{(t)}) - S(M_G^{(t)}) = C + \sum_{i=1}^{n} \log \mathbb{P}(D_i^{(t)}|\hat{\theta}, M_P^{(t)}) - \sum_{i=1}^{n} \log \mathbb{P}(D_i^{(t)}|\hat{\theta}, M_G^{(t)}),$$

    where, $C = 2p\log(n)$ if SC, or $C = 2p$ if AIC, and $\hat{\theta}$ is the maximum likelihood estimate of θ
8:     **if** $S(M_P^{(t)}) < S(M_G^{(t)})$, **then**
9:         Set $M_G^{(t+1)} = M_P^{(t)}$,
10:     **else**
11:         Set $M_G^{(t+1)} = M_G^{(t)}$
12:     **end if**
13:     Set $t = t + 1$
14: **end while**

We choose $M_G^{(0)}$ randomly with equal probability from models in $\mathcal{M}$. Θ determines $\theta_{min}, \theta_{max}$ and θ_T is chosen uniformly randomly on this interval. The parameters of the categorical distribution used in step 4 is determined by the proportion of scientists in the population.
