# Supplementary material for: Scientific discovery in a model-centric framework: Reproducibility, innovation, and epistemic diversity
Source: PLoS One. 2019 May 15;14(5):e0216125. doi: 10.1371/journal.pone.0216125 (PMC6519896; doi:10.1371/journal.pone.0216125)
Supplement: S1 Code and Data — (PDF) [file pone.0216125.s002.pdf]

Code and Data.

The code to perform the simulations and analyze the data generated in this project, and a summary data set are included as a Git repository at `https://github.com/gnardin/CRUST`. Refer to the description in the main page of this repository for further instructions and details of the implementation.
