# Supplementary material for: Scientific discovery in a model-centric framework: Reproducibility, innovation, and epistemic diversity
Source: PLoS One. 2019 May 15;14(5):e0216125. doi: 10.1371/journal.pone.0216125 (PMC6519896; doi:10.1371/journal.pone.0216125)
Supplement: S1 File — (PDF) [file pone.0216125.s019.pdf]

A stochastic process of scientific discovery.

Consider an infinite population of scientists conducting a sequence of idealized experiments $\xi^{(t)} := (M_P^{(t)}, \theta, D^{(t)}, S, K^{(t)})$, indexed by time $t = 1, 2, \cdots$ where $M_P^{(t)}$ belongs to a set of probability structures $\mathcal{M} = \{M_1, M_2, \cdots, M_L\}$ known to all scientists. Further, assume that there are A distinct scientist types in the population, each with a well-defined research strategy $R \in \mathcal{R} = \{R_o, R_1, \cdots, R_A\}$ of proposing a model in their experiment. These strategies depend on the type of scientist and a global model $M_G^{(t)} \in \mathcal{M}, K^{(t)}$, which represents the consensus of the scientist population at time t. The population of scientists aims to find the true model $M_T \in \mathcal{M}$. A scientist selected to conduct an experiment at time t, uses her background knowledge $K^{(t)}$ to propose a new candidate model $M_P^{(t)}$. Specifically, we define $K^{(t)}$ as a probability distribution $\mathbb{P}(M_P|R^{(t)}, M_G^{(t)})$, where $\{M_P, M_G^{(t)}\} \in \mathcal{M}^2$, and $R^{(t)} \in \mathcal{R}$.

The initial conditions of our stochastic process include the true model M_T, true parameter values θ_T of M_T, an initial global model $M_G^{(0)}$, a method for model selection S, and the sample size of the data n. At each time step, an idealized experiment $\xi^{(t)}$ is performed and new data $D^{(t)}$ of size n is generated independent of everything else from distribution $M_T(\theta_T)$. Each experiment is performed by a scientist randomly selected from A types in the population using the categorical distribution with probabilities $(p_1, p_2, \cdots, p_A)$. The selected scientist proposes a model $M_P^{(t)}$ with probability $\mathbb{P}(M_P|R^{(t)}, M_G^{(t)})$ conditional on a research strategy fully specified by her type and the current global model. Given the data $D^{(t)}$, model scores under the proposed model and the current global model are calculated as $S(M_P^{(t)})$ and $S(M_G^{(t)})$, respectively. The model with favorable score (i.e., smaller for both AIC and SC) is set as the new global model $M_G^{(t+1)}$. This mechanism represents how scientific consensus is updated in light of new evidence.

A defining property of our stochastic process with no replication is that $K^{(t)}$ depends only on quantities at time t. If $R_a \in \mathcal{R}$ depends only on $M_G^{(t)}$ for all a, the transition from $M_G^{(t)}$ to $M_G^{(t+1)}$ admits the Markov property and the stochastic process representing the scientific process is a Markov chain with transition probabilities given by

$$\mathbb{P}(M_G^{(t+1)} = M_\ell | M_G^{(t)} = M_i) = \sum_{a=1}^{A} \mathbb{P}(S(M_\ell) < S(M_i))\mathbb{P}(M_\ell|R_a, M_i)\mathbb{P}(R_a). \tag{1}$$

On the right hand side of Eq. (1), the last term is the probability of selecting a scientist with research strategy R_a independent of all else, the middle term is the

probability of proposing the model M_ℓ given the current global model M_i and the scientist type a with research strategy R_a selected. The probability $\mathbb{P}(S(M_\ell) < S(M_i))$ depends on M_T via $D^{(t)}$ generated and it is obtained by $\int_\Theta \int_\mathcal{D} \mathbb{P}(S(M_\ell) < S(M_i)|D)\mathbb{P}(D|\theta)\mathbb{P}(\theta)dDd\theta$, where $\mathbb{P}(\theta)$ is the probability of parameter, $\mathbb{P}(D|\theta)$ is the likelihood of the data, and $\mathbb{P}(S(M_\ell) < S(M_i)|D)$ is the probability that the proposed model M_ℓ has a more favorable score than M_i conditional on data. We have $\mathbb{P}(S(M_\ell) = S(M_i)) = 1$ when $\ell = i$ and the model selection method S is a continuous variable so that $\mathbb{P}(S(M_\ell) \leq S(M_i)) = \mathbb{P}(S(M_\ell) < S(M_i))$ and by convention we set $\mathbb{P}(S(M_\ell) < S(M_i)) = 1$. Further, $\mathbb{P}(M_\ell|R_a, M_i) > 0$ for all a, i, ℓ so that transition probabilities are nonzero for all models and scientist types. This second condition guarantees that our Markov chain is ergodic, which implies that it has a unique stationary distribution—its limiting distribution for visiting a model.

When there are no replication experiments in the system, $K^{(t)}$ is defined as $\mathbb{P}(M_P|R^{(t)}, M_G^{(t)})$ which states that conditional on $R^{(t)}$ and $M_G^{(t)}$, the probability of proposing a model is independent of the past time steps. Let $R_o \in \mathcal{R}$ be the replicator strategy. Given the proposed and global models at time $t-1$, the replicator strategy at time t, $R_o^{(t)}$, is to perform an experiment at time t, using the exact same proposed and global models as those at time $t-1$, but with new data $D^{(t)}$ generated under $M_T(\theta_T)$. Since $R_o \in R$ depends on $M_G^{(t-1)}$, the transition from $M_G^{(t)}$ to $M_G^{(t+1)}$ does not admit the Markov property anymore and the stochastic process representing the scientific process is a higher order Markov chain. The transition probabilities of the Markov chain at time t can be expressed by conditioning on whether a scientist chosen at a given time is a replicator:

$$\begin{aligned}
&\mathbb{P}(R^{(t)} \neq R_o)\mathbb{P}(M_G^{(t+1)}|M_G^{(t)}) + \\
&\mathbb{P}(R^{(t)} = R_o)[\mathbb{P}(R^{(t-1)} \neq R_o)\mathbb{P}(M_G^{(t+1)}|M_G^{(t)}, M_G^{(t-1)}) + \cdots + \\
&\mathbb{P}(R^{(1)} = R_o)[\mathbb{P}(R^{(0)} \neq R_o)\mathbb{P}(M_G^{(t+1)}|M_G^{(t)}, M_G^{(t-1)}, \cdots, M_G^{(0)})] \cdots]. \quad (2)
\end{aligned}$$

In Eq. (2), the first term in the sum is the joint probability of choosing a scientist who is not a replicator at time t and the transition probability from global model at time t to global model at time $t+1$. Since the scientists are chosen independently of all else, the joint probability is written as the product of choosing a scientist who is not a replicator at time t, given by $\mathbb{P}(R^{(t)} \neq R_o)$, and the probability of transition to the global model at time $t+1$ is given by Eq. (1). The second term in the sum is the joint probability of choosing a scientist who is a replicator at time t and the transition probabilities to a model. We write the second term as the product of $\mathbb{P}(R^{(t)} = R_o)$ and the transition probabilities when a replicator is chosen. If the scientist at time t is a replicator, she replicates the experiment at time

step $t-1$, which might be a replication experiment itself. Therefore, the transition probabilities to a model within the first brackets is a sum of two probabilities. The first term is the joint probability of choosing a scientist who is not a replicator and the transition probability in that case, and the second term is the probability of choosing a replicator given by $\mathbb{P}(R^{(t-1)} = R_o)$ at time step $t-1$, and the transition probability in that case. This is a recursive equation, in the sense that the transition probabilities at time t depend on the transition probabilities at time $t-1$. An implication is that the transition probabilities at time t are path dependent. Therefore, when a replicator scientist is included in the population, we have a higher order Markov chain, whose long term dynamics are feasible to obtain with a forward simulation method.

For the process with replicator, we lift the assumption $\mathbb{P}(M_\ell|R_a, M_i) > 0$ for all a, i, ℓ that we imposed in the process without a replicator. This assumption increases the connectivity of the transition probability matrix, which makes calculations in the long-term behavior of the Markov chain straightforward. Due to our new process not admitting the Markov property, these calculations are irrelevant in the analysis of the process with a replicator. Therefore, we drop the assumption of transitioning from a model to any other model to be nonzero. Removing this assumption allows us to define scientist types that visit only the subset of all models consistent with a specific research strategy. This property of the process renders the effects of each research strategy on the process outcomes well-pronounced.
