# Supplementary material for: Scientific discovery in a model-centric framework: Reproducibility, innovation, and epistemic diversity
Source: PLoS One. 2019 May 15;14(5):e0216125. doi: 10.1371/journal.pone.0216125 (PMC6519896; doi:10.1371/journal.pone.0216125)
Supplement: S2 File — (PDF) [file pone.0216125.s020.pdf]

Description of example system of linear models.

We define $\mathcal{M}$, the family of linear models as

$$\{\mathbf{y} = \mathbf{X}\boldsymbol{\beta} + \boldsymbol{\epsilon} | \beta_{i\cdots jv} \neq 0 \Rightarrow (\beta_i \neq 0, \cdots, \beta_v \neq 0, \cdots, \beta_{i\cdots j} \neq 0)\}.$$

Here, $\mathbf{y}$ is $n \times 1$ vector of response variables, $\mathbf{X}$ is $n \times p$ matrix of predictor variables, $\boldsymbol{\beta}$ is $p \times 1$ vector of model parameters, and $\boldsymbol{\epsilon}$ is $n \times 1$ vector of random errors satisfying the Gauss-Markov conditions $\mathbb{E}(\epsilon_i) = 0, \mathrm{Var}(\epsilon_i) = \sigma^2$, and $\mathrm{Cov}(\epsilon_i, \epsilon_j) = 0$ for all i, j. $\mathcal{M}$ contains L linear models with up to k factors subject to the constraint $\beta_{i\cdots jv} \neq 0 \Rightarrow (\beta_i \neq 0, \ \cdots, \beta_{i\cdots j} \neq 0)$ which guarantees that if a model contains a v–way $(v \leq k)$ interaction term of v factors in the model, then all $(v-1)$–way, $(v-2)$–way, $\cdots$, 2–way interactions of those factors and their main effects are included in the model. We include the predictor x_1 and the response variable y in all models reflecting our assumption that all scientists in the community focus on a research question that involves at least one common factor of interest and a common response variable. Let $M_i \in \mathcal{M}$ be a model with p_i parameters of which v_{ℓ_i} is the highest order interaction term with order ℓ_i denoting the order, $\#v_{\ell_i}$ is the cardinality of v_{ℓ_i}. Let $M_i \succ M_j$ denote that M_i is more complex than M_j. We define the *model complexity* as a partial ordering obeying three conditions:

1. If $p_i > p_j$ then $M_i \succ M_j$.
2. If $p_i = p_j$ and $\ell_i > \ell_j$, then $M_i \succ M_j$.
3. If $p_i = p_j, \ \ell_i = \ell_j$, and $\#v_{\ell_i} > \#v_{\ell_j}$, then $M_i \succ M_j$.

Otherwise, we say that complexities of M_i and M_j are indistinguishable.
