# Supplementary material for: Scientific discovery in a model-centric framework: Reproducibility, innovation, and epistemic diversity
Source: PLoS One. 2019 May 15;14(5):e0216125. doi: 10.1371/journal.pone.0216125 (PMC6519896; doi:10.1371/journal.pone.0216125)
Supplement: S3 File — (PDF) [file pone.0216125.s021.pdf]

Properties of scientific discovery.

How quickly does scientific community discover the true model? When there are no replicators in the model, $\mathbf{P} = \{\mathbb{P}(M_\ell|M_i)\}$ is the transition probability matrix given by Eq.(1) in S1 File. We assess the speed with which scientific community discovers the true model by the mean first passage (or hit) time to the true model. This is the first time to reach M_T, given that we start the system from M_i. The mean first passage time to the true model which we denote by $\tau_{i,T}$, is the expected value of this first passage time to select the true model as the global model for the first time in the process, given that the process starts from a known model M_i. By theory of Markov chains we have

$$\tau_{i,T} = 1 + \sum_{\substack{\ell=1 \\ M_\ell \neq M_T}}^{L} \mathbb{P}(M_\ell|M_i)\tau_{\ell,T},\ i = 1, 2, \cdots, L.$$

Given the transition probabilities $\mathbb{P}(M_\ell|M_i)$, the solution to this system of L linear equations is readily obtained by a method to solve linear equations.

When there are replicators in the model, we assess the speed to discover the true model by the mean number of steps for the system to update the global model to the true model for the first time. The first the time true model is global model is the mean first passage time to the true model described in the process of scientific discovery with no replication, but it is unconditional on the starting model.

How "sticky" is the true model as global model? We define stickiness of a model as the mean—over proposed models—probability of staying in model M_i conditional on the current global model M_i. When there are no replicators in the model, by theory of Markov chains, it is given by

$$\mathbb{P}(M_G^{(t+1)} = M_i|M_G^{(t)} = M_i) = \sum_{a=1}^{A}\sum_{\ell=1}^{L} \mathbb{P}(S(M_i) < S(M_\ell))\mathbb{P}(M_\ell|R_a, M_i)\mathbb{P}(R_a), \tag{1}$$

where $\mathbb{P}(R_a)$ and $\mathbb{P}(M_\ell|R_a, M_i)$ are same as in Eq.(1) in S1 File, and $\mathbb{P}(S(M_i) < S(M_\ell))$ is the probability that the current global model M_i has a more favorable score than the proposed model M_ℓ. The right hand side of Eq. (1) can be calculated for all L models given M_T. This probability captures the tendency of scientific community to stay at a visited model. Calculated for $M_G^{(t+1)} = M_G^{(t)} = M_T$, it gives the stickiness of the true model.

When there are replicators in the model, we measure the tendency of the scientific community to maintain consensus on the true model by the proportion of time that true model remains as global model given that it was already the global model.

Stickiness is the complement of the probability that a global model switches from true model to another model.

How long does scientific community stay on the true model? By theory of ergodic Markov chains, the probability that each model is selected as the global model converges to a constant value independent of the current global model $M_G^{(t)}$. The limiting probabilities of a model being selected as a global model reflect the long-term behavior of the scientific community and the proportion of time spent on each model. These limiting probabilities are given by $\lim_{t\to\infty} \mathbf{P}^t$.

When there are replicators in the model, we assess the mean time the scientific community spends on a model (and in particular the true model) as a consensus by the proportion of times that a model is selected as the global model. We use the proportion of times true model is selected as global model as a proxy for the limiting probabilities of selecting the true model as the global model in the process of scientific discovery with no replication.

How reproducible are the results of experiments? Under ABM, we define the rate of reproducibility as the expectation of Bernoulli distributed random variable $\mathbf{I}_{\{M_G^{(t+1)}=M_G^{(t)}|R_o^{(t)}\}}$, which is the indicator function that takes the value 1 if the global model at time $t+1$ is the same as the global model at t given that a replication experiment is performed at time t. The parameter of the Bernoulli distribution is the probability of reproducibility $\mathbb{P}(M_G^{(t+1)} = M_G^{(t)}|R_o^{(t)})$. Further, we define the rate of reproducibility when the true model is the global model by $\mathbf{I}_{\{M_G^{(t+1)}=M_G^{(t)}|R_o^{(t)},M_G^{(t)}=M_T\}}$ which also determines the rate of reproducibility when the true model is not the global model $\mathbf{I}_{\{M_G^{(t+1)}=M_G^{(t)}|R_o^{(t)},M_G^{(t)}\neq M_T\}}$. We estimate these rates of reproducibility by Monte Carlo integration of ABM simulations.

Monte Carlo estimates of rates of reproducibility

The random variable $\mathbf{I}_{\{M_G^{(t+1)}=M_G^{(t)}|R_o^{(t)}\}}$ which takes the value 1 if the global model at time $t+1$ is equal to global model at time t given that at time t we have chosen a replicator, is a Bernoulli distributed random variable. Its mean is given by $\mathbb{E}(\mathbf{I}_{\{M_G^{(t+1)}=M_G^{(t)}|R_o^{(t)}\}})$ whose Monte Carlo estimate is given by

$$\widehat{\mathbb{E}}(\mathbf{I}_{\{M_G^{(t+1)}=M_G^{(t)}|R_o^{(t)}\}}) = \frac{1}{V}\sum_{v=1}^{V} \mathbf{I}_{\{M_G^{(t+1)}=M_G^{(t)}|R_{ov}^{(t)}\}},$$

where $R_{o_v}^{(t)}$ is the vth instance a replicator is chosen. The rate of reproducibility when the true model is global model is estimated by

$$\widehat{\mathbb{E}}(\mathbf{I}_{\{M_G^{(t+1)}=M_G^{(t)}|R_0^{(t)},M_G^{(t)}=M_T\}}) = \frac{1}{V_T}\sum_{v=1}^{V_T}\mathbf{I}_{\{M_G^{(t+1)}=M_G^{(t)}|R_{0_v}^{(t)},M_G^{(t)}=M_T\}}$$

which also implies

$$\widehat{\mathbb{E}}(\mathbf{I}_{\{M_G^{(t+1)}=M_G^{(t)}|R_0^{(t)},M_G^{(t)}\neq M_T\}}) = \frac{1}{V_N}\sum_{v=1}^{V_N}\mathbf{I}_{\{M_G^{(t+1)}=M_G^{(t)}|R_{0_v}^{(t)},M_G^{(t)}\neq M_T\}}.$$

Here, V_T is the number of times a replicator is selected when the true model is global model and V_N is the number of times a replicator is selected when the true model is not global model, and $V_T + V_N = V$.
