# Supplementary material for: Scientific discovery in a model-centric framework: Reproducibility, innovation, and epistemic diversity
Source: PLoS One. 2019 May 15;14(5):e0216125. doi: 10.1371/journal.pone.0216125 (PMC6519896; doi:10.1371/journal.pone.0216125)
Supplement: S4 File — (PDF) [file pone.0216125.s022.pdf]

Properties of the model.

Predictor variables, error variance, and sample size. We uniformly randomly generate the value of the jth factor at the ith level x_{ij} on the set $\{1, 2, \cdots, 100\}$ for all i and j. We fix the sample size $n = 100$ and calibrate the ratio of the error variance σ^2 to expected value of the model at the mean value of the predictors $\mathbb{E}(y|\mu_x)$, where $\mu_x = \mathbb{E}(x)$. We use a standardized linear regression model so that if the ith observed response is y_i^*, then we set $y_i = (y_i^* - \bar{y}^*)/s^*$, where $\bar{y}^*$ is the sample mean and s^* is the sample standard deviation of $y_i^*,\ i = 1, 2, \cdots, n$. Implementing standardized linear regression model allows us to precisely specify $\sigma^2 : \mathbb{E}(y|\mu_x)$. We fix three levels for $\sigma^2 : \mathbb{E}(y|\mu_x)$. $(1:4)$, $(1:1)$, and $(4:1)$. We set the true regression coefficients to be Dirichlet distributed with unit parameters or equivalently, uniformly distributed on the interval $(0, 1)$ with the constraint that they sum to 1. Thus, all regression coefficients have the same mean effect size. We set the correlation between the first factor x_1 and other factors to a small value of 0.2 to avoid orthogonality between predictors which is an idealized case that we believe rarely achievable in practice. We performed additional analyses with higher correlation between predictor variables and found that correlation between predictors does not affect the results in our system unless it is extremely high and causes multicollinearity.

Model selection criteria. SC is defined by $p\log(n) - \log\mathbb{P}(D|\hat{\theta}, M)$, where p is the number of model parameters, n is the sample size, and $\hat{\theta}$ is the maximum likelihood estimate of model parameters under M. Akaike's Information Criterion (AIC) is defined by $2p - \log\mathbb{P}(D|\hat{\theta}, M)$. For both SC and AIC, a smaller value indicates a better model performance than a larger value. The maximized likelihood $\log\mathbb{P}(D|\hat{\theta}, M)$ rewards model fit equally in SC and AIC. The first term in these formulas penalize the model complexity, with SC penalizing complex models more heavily for $n \geq 8$ (with $\log(n)$) than AIC as the sample size increases. SC has the desirable property that when the true model generating the data is in the universe of candidate models, it selects the true model with probability 1 as $n \to \infty$, in other words it is consistent. For model M fitted to D, we calculate the model selection criteria SC and AIC in a computationally efficient way.

Description of scientists and scientist populations. We let

$$\mathcal{R} = \{R_{Rey}, R_{Tess}, R_{Mave}, R_{Bo}\}$$

and define the transition probabilities for these strategies as follows. For R_{Rey},

$$\mathbb{P}(M_P^{(t)} = M_j | M_P^{(t-1)} = M_i, M_G^{(t)} = M_G^{(t-1)}) = \begin{cases} 1 & \text{if } i = j, \\ 0 & \text{otherwise.} \end{cases}$$

For R_{Tess},

$$\mathbb{P}(M_P^{(t)} = M_i | M_G^{(t)}) = \begin{cases} 1/(m+1) & \text{if } M_i \text{ is one of } m \text{ models} \\ & \text{one term away from } M_G^{(t)}, \\ m_o & \text{otherwise.} \end{cases}$$

If $R_{Rey} \in \mathcal{R}$ then $m_o = 0$ and $m_o = 1/[(L-m)(m+1)]$ otherwise. For R_{Mave}, $\mathbb{P}(M_P^{(t)} = M_i | M_G^{(t)}) = 1/L,\ i = 1, 2, \cdots, L$. For R_{Bo},

$$\mathbb{P}(M_P^{(t)} = M_i | M_G^{(t)}) = \begin{cases} 1/(m+1) & \text{if } M_i \text{ is one of } m \text{ models one} \\ & \text{interaction larger than } M_G^{(t)}, \\ m_o & \text{otherwise.} \end{cases}$$

If $R_{Rey} \in \mathcal{R}$ then $m_o = 0$ and $m_o = 1/[(L-m)(m+1)]$ otherwise.
