# Supplementary material for: Scientific discovery in a model-centric framework: Reproducibility, innovation, and epistemic diversity
Source: PLoS One. 2019 May 15;14(5):e0216125. doi: 10.1371/journal.pone.0216125 (PMC6519896; doi:10.1371/journal.pone.0216125)
Supplement: S5 File — (PDF) [file pone.0216125.s023.pdf]

Monte Carlo estimates of model comparisons.

Let $\mathbf{y}$ denote $n \times 1$ vector of responses generated by the true model and $\mathbf{X}$ be $n \times p$ matrix of predictors, where p is the number of parameters in the fitted model. Under the Gauss-Markov assumptions $\mathbb{E}(\epsilon_i) = 0,\ Var(\epsilon_i) = \sigma^2,\ Cov(\epsilon_i, \epsilon_j)$ for all i, j we denote the vector of joint maximum likelihood estimates for the regression coefficients by $\hat{\boldsymbol{\beta}}$. By definition of Schwarz Criterion

$$S(M) = 2p\log(n) - 2\log\mathbb{P}(\mathbf{y}|\mathbf{X}, \hat{\boldsymbol{\beta}}).$$

If Akaike's Information Criterion is used, $2p$ replaces $2p\log(n)$. The loglikelihood in the second term is equal to n times the log of the residual sums of squares and it can be written as

$$\log\mathbb{P}(\mathbf{y}|\mathbf{X}, \hat{\boldsymbol{\beta}}) = n\log(\mathbf{y}'\mathbf{y} - \mathbf{y}'\mathbf{X}(\mathbf{X}'\mathbf{X})^{-1}\mathbf{X}'\mathbf{y}) + C,$$

where C is a term dependent only on M_T. For transition probabilities, we are interested in $\mathbb{P}(S(M_i) < S(M_j))$. We have

$$\begin{aligned} S(M_i) - S(M_j) &= (p_i - p_j)\log(n) + n\log(\mathbf{y}'\mathbf{X}_i(\mathbf{X}_i'\mathbf{X}_i)^{-1}\mathbf{X}_i'\mathbf{y}) \\ &\quad -n\log(\mathbf{y}'\mathbf{X}_j(\mathbf{X}_j'\mathbf{X}_j)^{-1}\mathbf{X}_j'\mathbf{y}), \qquad (1) \end{aligned}$$

where subscripts i, j now denote quantities that depend on model M_i and M_j. The random variable $\mathbf{I}_{\{S(M_i)-S(M_j)<0|M_T\}}$ is Bernoulli distributed with probability of success $\mathbb{P}(S(M_i) - S(M_j) < 0)$ which is equal to its expectation $\mathbb{E}(\mathbf{I}_{\{S(M_i)-S(M_j)<0|M_T\}})$ whose Monte Carlo estimate is given by

$$\widehat{\mathbb{E}}(\mathbf{I}_{\{S(M_i)-S(M_j)<0|M_T\}}) = \frac{1}{V}\sum_{v=1}^{V}\mathbf{I}_{\{S(M_i)-S(M_j)<0|\mathbf{y}_v\}}. \qquad (2)$$

An estimate of $\mathbb{P}(S(M_i) - S(M_j) < 0)$ is obtained using Eq. (2) conditional on true model M_T and its predictors X_T as follows. First, generate the set of k predictor variables and build $\mathbf{X}_i$ and $\mathbf{X}_j$ for M_i and M_j respectively. Then generate $\boldsymbol{\beta}_{\boldsymbol{T_v}},\ v = 1, 2, \cdots, V$ independently of each other. Finally, simulate $\mathbf{y}_v|\mathbf{X}_T, \boldsymbol{\beta}_{\boldsymbol{T_v}}$ from the normal distribution with expected value $\mathbb{E}(\mathbf{y}_v) = \mathbf{X}_T\boldsymbol{\beta}_{\boldsymbol{T_v}}$ and variance σ^2. Each realization $(\mathbf{y}_1, \mathbf{y}_2, \cdots, \mathbf{y}_v)$ is used in Eq. (1) to assess $S(M_i) - S(M_j) < 0$ and the estimate is obtained using the mean in Eq. (2).
