# Supplementary material for: Scientific discovery in a model-centric framework: Reproducibility, innovation, and epistemic diversity
Source: PLoS One. 2019 May 15;14(5):e0216125. doi: 10.1371/journal.pone.0216125 (PMC6519896; doi:10.1371/journal.pone.0216125)
Supplement: S6 File — (PDF) [file pone.0216125.s024.pdf]

Reproducibility does not imply discovery of truth.

In this section, we show that the rate of reproducibility has no causal effect on other desirable properties of scientific discovery including: the probability that a model is selected as the global model in the long run, the mean first time to hit a model, and stickiness of a model. Our conclusion follows directly from our definition of reproducibility of a result being conditional on the result of the experiment immediately before that (after performing a deliberate replication experiment in the second step). In Baumgaertner et al. (2018), we distinguish between two types of reproducibility–*in-principle* and *epistemic*–and discuss how these two types can arise. The part that is relevant to our illustration here is that even if a result is independently obtained in multiple experiments, unless there is a flow of knowledge about the results of these experiments, it is not possible to claim *epistemic* reproducibility. For our system, we do not consider or track *in-principle* reproducibility and assume that reproducibility is realized if and only if an experiment deliberately repeats a previous experiment with known results, obtains the same result as the previous experiment, and can claim that the result is reproduced. This makes reproducibility a conditional event based on two experiments. The result present here about the rate of reproducibility having no causal effect on other desirable properties of scientific discovery should be interpreted in this context. This result can be generalized to conditioning reproducibility on multiple experiments and we mention this case below, but do not include the calculations which add little to our point.

The key observation for the result is that all of these desirable properties of scientific discovery are properties of the stochastic process of scientific discovery defined by the Markov chain. A Markov chain is characterized solely by its transition probability matrix. Below, we show that the rate of reproducibility, does not affect the transition probability matrix in our model. Therefore, any property of the Markov chain is not affected by the rate of reproducibility. To keep the notation tractable and without loss of generality we assume that *Rey*, the replicator is not chosen consecutively in the process. Generalization to the case where *Rey* is chosen consecutively is by induction and using Eq.(2) in S1 File.

When *Rey*, the replicator, is in the system, the model is a second order Markov chain, which has transitions specified over two time steps. We let the triplet of indices (i, j, ℓ) to be associated with a transition in this second order Markov chain, where i is the beginning state and ℓ is the final transition state through state j. We let R_{Rey} to be the replicator strategy and $R_{Rey'}$ to be any non-replicator strategy. $P(R_{Rey}) = 1 - P(R_{Rey'})$ is the probability that the second step is a replication experiment. The probability that the global model transitions to M_ℓ in the second

step given that M_i was the beginning global model can be written as

$$P(R_{Rey'}) \sum_{j=1}^{L} p_{ij\ell} + P(R_{Rey}) \sum_{j=1}^{L} q_{ij\ell}, \qquad (1)$$

where $p_{ij\ell}$ and $q_{ij\ell}$ are transition probabilities when a replicator is not selected and is selected, respectively. The first term in Eq. (1) does not involve a replicator so to establish whether the rate of reproducibility affects transition probabilities it is sufficient to focus on the second term. There are only two nonzero terms in $\sum_{j=1}^{L} q_{ij\ell}$. The first term is

$$q_{ii\ell} = P(S(M_\ell) < S(M_i))P(S(M_\ell) < S(M_i))P(M_\ell|M_i),$$

where on the right hand side: M_ℓ is proposed with probability $P(M_\ell|M_i)$ and wins against M_i with probability $P(S(M_\ell) < S(M_i))$ at the first step, and in the replication (second step) it wins again with probability $P(S(M_\ell) < S(M_i))$ to stay as the global model. The case of $\ell = i$ is included in this first term because $P(S(M_\ell) < S(M_i)) = 1$ for $\ell = i$ by convention as defined in S1 File, which means that the model M_i wins in the first step and in the second step (against itself in both steps). The second term nonzero term is

$$q_{i\ell\ell} = P(S(M_\ell) < S(M_i))P(S(M_\ell) > S(M_i))P(M_\ell|M_i),$$

where on the right hand side: M_ℓ is proposed with probability $P(M_\ell|M_i)$ and loses against M_i with probability $P(S(M_\ell) > S(M_i))$ at the first step, and in the replication (second step) it wins against M_i with probability $P(S(M_\ell) < S(M_i))$ to become the global model. Here, $q_{ii\ell}$ and $q_{i\ell\ell}$ are the probabilities of reproducing and not reproducing a result, respectively. Further, only their sum contributes to the Markov transition probability matrix (Eq. (1)). We write

$$\begin{aligned}\sum_{j=1}^{L} q_{ij\ell} &= q_{ii\ell} + q_{i\ell\ell} \\ &= P(S(M_\ell) < S(M_i))P(S(M_\ell) < S(M_i))P(M_\ell|M_i) \\ &+ P(S(M_\ell) < S(M_i))P(S(M_\ell) > S(M_i))P(M_\ell|M_i) \\ &= P(S(M_\ell) < S(M_i))P(M_\ell|M_i)[P(S(M_\ell) < S(M_i)) + P(S(M_\ell) > S(M_i))],\end{aligned}$$

where the first and second term within brackets determine whether a result is reproduced or not reproduced, respectively, and sum to 1. Therefore we have

$$\sum_{j=1}^{L} q_{ij\ell} = P(S(M_\ell) < S(M_i))P(M_\ell|M_i). \qquad (2)$$

This last equation states that the transition probabilities depend on $P(S(M_\ell) < S(M_i))$ which is the probability of M_ℓ winning in the second experiment against M_i and $P(M_\ell|M_i)$ which is the probability of proposing M_ℓ given that the global model is M_i in the first step. In particular, we note that because reproducibility is defined as a conditional event based on two steps and $P(S(M_\ell) < S(M_i))$ concerns only a comparison of models of the second of these steps, the information about whether a result is reproduced or not is not conserved. Consequently, whether a result is reproduced has no bearing on the calculation of transition probability from M_i to M_ℓ in our system because the results from the first step are summed over. If we had defined reproducibility as obtaining the same results based on two replication experiments and an original experiment, the Markov chain now would be defined as order three and the first and second step results would be summed over, again yielding transition probabilities independent of whether a result is reproduced or not. Since the transition probability matrix characterizes a Markov chain, the rate of reproducibility cannot affect other desirable properties of scientific discovery including: the probability that a model is selected as the global model in the long run, the mean first time to hit a model, and the stickiness of a model.

On the other hand, we do not claim that there cannot be correlation between desirable properties of scientific discovery and the rate of reproducibility. In fact, whether there is correlation depends on the strategies and their frequency in the population, and this also can be seen from our mathematical result. For transition to M_ℓ the rate of reproducibility is proportional to $\sum_{i \neq \ell} P(S(M_\ell) < S(M_i))$ and the transition probability to M_ℓ is proportional to $P(S(M_\ell) < S(M_i))$. If the effect of the first term $P(R_{Rey'}) \sum_{j=1}^{L} p_{ij\ell}$ in Eq. (1) and the second term $P(M_\ell|M_i)$ in Eq. 2 are small then there might be considerable correlation between desirable properties of scientific discovery and the rate of reproducibility. This relationship depends on a number of factors including the exact strategies of scientists in the population and the frequency of these scientists. The code to perform the simulations and analyze the data generated in this project, and a summary data set are included as a Git repository at `https://github.com/gnardin/CRUST`. Refer to the description in the main page of this repository for further instructions and details of the implementation.
