# Supplementary material for: Scientific discovery in a model-centric framework: Reproducibility, innovation, and epistemic diversity
Source: PLoS One. 2019 May 15;14(5):e0216125. doi: 10.1371/journal.pone.0216125 (PMC6519896; doi:10.1371/journal.pone.0216125)
Supplement: S7 File — (PDF) [file pone.0216125.s025.pdf]

ABM with soft research strategies.

	Rey	Tess	Mave	Bo	All equal
Median	815	80	16	18	25
IQR	1686	620	36.25	40	46

Median and IQR of the mean (over 100 replications for each parameter set) first passage time to true model calculated over runs with different model parameters for ABM with soft research strategies. Mean first passage time to true model is faster for *Tess*- and *Bo*-dominant populations when scientists in the ABM simulations pursue soft (vs. hard) research strategies, consistent with results from Markov chain without replicator. The median of the mean first passage time for *Mave*-dominant population is the same as the median we reported for ABM with hard research strategies because the maverick strategy has a well connected transition matrix, leading to quick discovery of the true model. Further, the representation of *Mave*s in the epistemically diverse population makes this population also well connected, in ABM with both hard and soft research strategies. *Rey*-dominant populations are the slowest to discover the truth because when replicators comprise 99% of the population, there are not many new models being proposed and same experiments are repeated many times slowing down the discovery of the true model.
