# Supplementary material for: Scientific discovery in a model-centric framework: Reproducibility, innovation, and epistemic diversity
Source: PLoS One. 2019 May 15;14(5):e0216125. doi: 10.1371/journal.pone.0216125 (PMC6519896; doi:10.1371/journal.pone.0216125)
Supplement: S1 Table — (PDF) [file pone.0216125.s026.pdf]

Populations of scientists with varying proportions of scientist types.

Scenario	Population	Rey	Mave	Tess	Bo
Without Replicator	Tess Dominant	0	0.005	0.99	0.005
	Mave Dominant	0	0.99	0.005	0.005
	Bo Dominant	0	0.005	0.005	0.99
	All Equal	0	$0.\bar{3}$	$0.\bar{3}$	$0.\bar{3}$
With Replicator	Rey Dominant	0.99	$0.00\bar{3}$	$0.00\bar{3}$	$0.00\bar{3}$
	Tess Dominant	$0.00\bar{3}$	$0.00\bar{3}$	0.99	$0.00\bar{3}$
	Mave Dominant	$0.00\bar{3}$	0.99	$0.00\bar{3}$	$0.00\bar{3}$
	Bo Dominant	$0.00\bar{3}$	$0.00\bar{3}$	$0.00\bar{3}$	0.99
	All Equal	0.25	0.25	0.25	0.25
