# Supplementary material for: Scientific discovery in a model-centric framework: Reproducibility, innovation, and epistemic diversity
Source: PLoS One. 2019 May 15;14(5):e0216125. doi: 10.1371/journal.pone.0216125 (PMC6519896; doi:10.1371/journal.pone.0216125)
Supplement: S2 Table — (PDF) [file pone.0216125.s027.pdf]

Parameter values used in ABM experiment.

**Parameter**	**Value**
`replications`	100
`timesteps`	11000
`k`	3
`sigma`	0.2, 0.5 and 0.8
`sampleSize`	100
`trueModel`	`x1 + x2`,<br>`x1 + x2 + x3 + x1x2`, and<br>`x1 + x2 + x3 + x1x2 + x1x3 + x2x3`
`correlation`	0.2
`nRey`	1 and 300
`nTess`	1 and 300
`nBo`	1 and 300
`nMave`	1 and 300
`modelCompare`	`AIC` and `BIC`
`ndec`	4
