# Supplementary material for: Scientific discovery in a model-centric framework: Reproducibility, innovation, and epistemic diversity
Source: PLoS One. 2019 May 15;14(5):e0216125. doi: 10.1371/journal.pone.0216125 (PMC6519896; doi:10.1371/journal.pone.0216125)
Supplement: S3 Table — Spearman rank-order correlation coefficients between rate of reproducibility and other desirable properties of scientific discovery for each scientist population. Overall is averaged over all scientist populations. (PDF) [file pone.0216125.s028.pdf]

Correlations per scientist population.

r_{SR}	Overall	Rey	Tess	Mave	Bo	All equal
Time spent at true model	-0.02	0.41	0.35	0.69	-0.06	0.59
Mean first passage time to true model	0.26	0.03	0.023	-0.13	0.03	-0.06
Stickiness	0.55	0.74	0.87	0.76	-0.03	0.82
